# Association of various reproductive rights, domestic violence and marital rape with depression among Pakistani women

**DOI:** 10.1186/1471-244X-9-77

**Published:** 2009-12-01

**Authors:** Faridah A Ali, Syed M Israr, Badar S Ali, Naveed Z Janjua

**Affiliations:** 1 Department of Community Health Sciences, Aga Khan University, Karachi, Pakistan; 2 Department of Family Medicine, Aga Khan University, Karachi, Pakistan; 3 TACMIL Health Project, Islamabad, Pakistan; 4 British Columbia Centre for Disease Control, Vancouver, Canada

## Abstract

**Background:**

Depression among women is common in developing countries. Gender inequality can contribute to women's risk for depression. Lack of reproductive and sexual rights is an important marker of gender inequality and women do not have the freedom to express their reproductive and sexual needs in many parts of the world. Therefore we designed this study to determine the association of depression with lack of various reproductive rights and domestic violence among married women in Karachi, Pakistan.

**Methods:**

A case-control study with 152 cases and 152 controls, which included women 15-48 years, recruited from two teaching hospitals from 1^st ^June 2007 through 31^st ^August 2007. The SRQ was administered to all subjects. A cut off score of 8 was used to confirm cases of depression diagnosed by physicians, and to exclude cases of depression from the controls. Self-administered questionnaire was used to assess the risk factors.

**Results:**

61% of the cases and 43% of the controls were ever abused by spouse and the frequency of marital rape was 33% in cases and 13% in controls. After adjusting for the effects of other variables in the model, less than 18 years of age at marriage (OR 2.00; 95% CI = 1.07, 3.7), decision for marriage by parents (OR 3.51; 95% CI = 1.67, 7.37), abuse by in laws (OR 4.91; 95% CI = 2.66, 9.06), ≤ 3 hours per day spent with husband (OR 2.33; 95% CI = 1.34, 4.08), frequency of intercourse ≤ 2 times per week (OR 1.85; 95% CI = 1.06, 3.22) and marital rape (OR 3.03; 95% CI = 1.50, 6.11) were associated with depression among women.

**Conclusion:**

In our study depression in married women was associated with younger age at marriage, lack of autonomy in marriage decisions, marital rape and domestic abuse by in-laws. Efforts should be directed towards creating awareness about the reproductive and sexual rights of women in Pakistan. Physicians should be trained to screen and identify women who may be at risk for psychological distress as a result of denial of reproductive rights so that they can support positive mental health outcomes through individual, family or marital counseling.

## Background

Reproductive rights rest on "the right of all couples and individuals to decide freely and responsibly the number, spacing and timing of their children, to have the information and means to do so, and the right to attain the highest standard of sexual and reproductive health; including the right to make decisions concerning sex and reproduction free of discrimination, coercion and violence". Gender discrimination is an important factor which deprives women of their reproductive rights [1].

Sexual rights are an important part of reproductive rights which include "The right to sexual freedom, autonomy, integrity, and safety of the sexual body. It also includes the right to sexual privacy, equity, sexual pleasure, emotional sexual expression, the right to sexually associate freely, right to make free and responsible reproductive choices, the right to sexual information, education and the right to sexual health care"[2].

In Pakistan, women are under-privileged in meeting their sexual needs and in freedom of choice of their partners, which has implications for women's reproductive behavior and human rights. This gender inequality decreases women's ability to have a healthy sex life, and increases their risk of violence and mental disorders [3-5].

Studies have found a correlation of depression with certain reproductive rights, such as younger age at marriage and marital rape [6-8]. Marital rape is any unwanted intercourse or penetration (vaginal, anal, or oral) obtained by force, threat of force, or when the wife is unable to consent [9]. Commonly it is perceived that women are raped by men other than their partners but data have revealed that, over 75% of the women who have been physically or sexually abused, report abuse by their partner. About 10-20% of the women surveyed in 5 out of 10 countries believed that a woman does not have a right to refuse sex to her husband under any circumstances [10]. It is now a crime in most parts of the Western world [9] but the Pakistani law still fails to recognize marital rape as a crime [11]. Although the association of depression with autonomy to decide about contraception has not been studied in the past, it is known that the stress of an unintended pregnancy or unsafe abortion might be expected to increase the risk of onset or recurrence of serious mental ill-health [12].

Domestic violence is also a risk factor for depression among women [7,13,14]. A study in Pakistan reported that 34% of women are physically abused, and of these 72% had anxiety/depression [15]. In another study in Pakistan, 95% men reported perpetrating verbal abuse during their marital life, which implies that verbal abuse by male partners is viewed as a norm [16]. Women also view a certain amount of physical abuse, as justified under certain conditions. For instance, 80% of women surveyed in rural Egypt said that beatings were common and often justified, particularly if the woman refused to have sex with her husband [17]. In a World Health Organization (WHO) report, studies conducted in 10 countries revealed that women who had ever experienced physical or sexual partner violence, or both, reported significantly higher levels of emotional distress and were more likely to have thought of suicide or to have attempted suicide, than were women who had never experienced partner violence [10].

Studies from Pakistan indicate that depression is common in women and reported various social and sociodemographic risk factors associated with depression in women [13,14,18-23]. However, the relationship between reproductive rights of women and depression has never been assessed. Exploring women's reproductive rights and their association with depression is more important in Pakistani context because of the specific culture, where women do not have control over their own reproduction [12] and disclosure of their sexual needs is difficult, and often stigmatized.

Reproductive rights is not only absence of reproductive illness but the right to life and health, rights to bodily integrity and security, the right to the benefits of scientific progress (e.g. control of reproduction), the right to sexual education, the right to equality in marriage and divorce and the right to non-discrimination [12]. Therefore this study aimed to determine the association of depression in married women in Pakistan with lack of various reproductive rights and other markers of gender inequality, like domestic violence.

## Methods

### Study design and study participants

A case control study was conducted at psychiatry and family medicine clinics of Aga Khan University Hospital (AKUH) and psychiatry clinics at Liaquat National Hospital (LNH) from 1^st ^June 2007 till 31^st ^August 2007.

Cases comprised of all consecutive currently married women, 15-48 years of age, attending the clinics mentioned above, who did not have a history of any other psychiatric illness except depression and were not on any antidepressants for the last 2 weeks. Cases were diagnosed as suffering with depression by psychiatrist or family physician according to DSM-IV (Diagnostic and Statistical Manual of Mental Disorders) which is a clinical criterion used to diagnose depression [24], and currently having a score of 8 or more on SRQ. Subjects were recruited from a clinic only if the psychiatrist or physician confirmed use of DSM-IV for the diagnosis of depression. They were also given the list of DSM-IV depression criteria by the research team, for convenient reference.

Controls were selected by convenience sampling, from among women 15-48 years of age who were either attendants accompanying the cases or any patient visiting the consulting clinics. These were currently married women of reproductive age, who did not have any psychiatric history or current depression according to SRQ 20 (score of 7 or less). Controls, who on screening had a score of 8 or more were also excluded and referred to a Family physician at AKUH for confirming the diagnosis and for further management. The other exclusion criteria for both cases and control were history of any chronic medical illnesses (Diabetes Mellitus, Hypertension, Ischemic heart disease, Chronic renal failure, Chronic liver disease, Rheumatoid arthritis, Thyroid disorders, Malignancy, Chronic obstructive pulmonary disease, Asthma). Subjects who were on any medication except micronutrients, pregnant/postpartum (up to 4 weeks of delivery) or post menopausal were also excluded.

### Questionnaire and data collection

A 20 item Self Reporting Questionnaire (SRQ 20) for anxiety and depression [25] was administered to all subjects. A cut off score of 8 was used to confirm cases of depression diagnosed by physicians, and to exclude cases of depression from the controls.

The SRQ was developed by Harding et al. (1980) for a WHO collaborative study to screen for common mental disorders in primary health care. The WHO formally recommended the SRQ 20 in its 1994 manual, which also reviewed the number of SRQ20 studies and reported the validity and reliability of the instrument. The sensitivity of SRQ ranges from 63-90% and specificity from 44-95% (tested against in-depth psychiatric interview) [25]. The validity of the SRQ has been established in Urdu in the Pakistani population as well [26].

Data were collected through a pretested (10% of sample size), self-administered questionnaire in English or Urdu (the lingua franca of Pakistan). If subjects were unable to read or write Urdu or English the questionnaire was verbally administered by an interviewer and confidentiality was ensured. The completed questionnaires were dropped in closed boxes in order to maintain their confidentiality.

Questionnaire included information on socio-demographic characteristics, and various items on reproductive rights. Questions on reproductive rights and domestic violence were designed based on previous research conducted in Pakistan and WHO multicountry study on women health and domestic violence [1,4,10]. Questionnaire included items on relationship with husband measured by inquiring about support provided in family conflicts and verbal, emotional and physical abuse, relationship with in-laws measured by inquiring, if living with livings, relationship (good, satisfactory or unsatisfactory) and verbal, emotional and physical abuse by in-laws. Items about marriage and current relationship included age at marriage, decision about the future partner, forced marriage, meeting with spouse before marriage, like spouse before marriage, number of years married, satisfaction with marriage, hours spent with husband per day, frequency of sexual intercourse per week, husband allowance of intimacy, satisfaction after intimacy and marital rape. Marital rape was assessed by asking women if their spouses have ever forced them (through beating, abusing or threatening), to have intercourse with them. Items on decision making about the contraception/pregnancy and children planning included family ever planned, number of children planned, decision on family planning, decision in case of disagreement, contraception use, and decision on contraception.

Our sample size calculation was based on findings from studies, that physical abuse by husband is a risk factor for depression [7,13], and 50% women in Pakistan are physically abused by their husbands [27]. It was therefore assumed that 50% (range 25-60%) of the non depressed women were physically abused by spouses (exposure among controls) with 5% probability of type 1 error and power of 80%, with an Odds ratio worth detecting of 2.0, the sample size was calculated as at least 298 with 149 cases and 149 controls. Taking range from 25-60%, the sample size was calculated as 152 cases and 152 controls [28]. To account for 10% non response in cases and 10% non response in controls, 168 cases and 168 controls were approached (Ratio 1:1).

The study was approved by the AKUH Ethical Committee. An informal verbal consent was taken by the consulting physician from the cases, and if they consented to participate then a formal written consent was taken by the data collectors.

### Statistical analysis

Data were entered in EPIDATA and analysis was performed using Statistical Package for the Social Sciences (SPSS) version 14. We compared distribution of variables between cases and controls by computing proportions for categorical variables and means and medians for quantitative variables. Categories of education, ethnicity, occupation and occupations were collapsed to obtain cell count appropriate for analysis.

Crude odds ratio and their 95% confidence interval (95% CI) were computed through logistic regression model developed for each independent variable. Risk factors with a p value of < 0.25 were considered for inclusion in the multivariate model. We started with the most significant variable and added variables one by one while assessing their significance and change in effect estimate. Variables that were not significant or did not produce change in effect estimate of > 10% were removed from the model. Variables significant at *P *< 0.05 were kept in the final model. The final model was tested for goodness of fit by the Hosmer Lenshow statistic.

## Results

**Figure 1:**
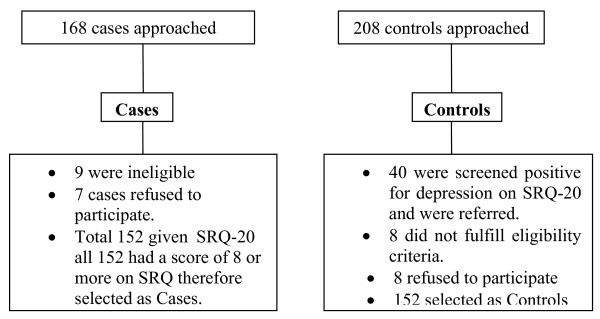
**Study participants**.

We enrolled 152 women of 15-48 years as cases, including 42 (27.6%) from Psychiatry clinics and 60 (39.5%) from Family medicine clinics of AKUH, and 50 (32.9%) from Psychiatry clinics of LNH with equal number of controls from each clinic. The number of controls had to be increased as on screening of controls 40 were found to have had a score of more than or equal to 8 on the SRQ and hence were referred to a family physician. The participation rate was 96%, and 100% of the participants answered all the questions in the questionnaire (Figure 1).

The mean age (SD) of controls was 31.0 (7.1) and of cases was 31.3 (7.5). A higher proportion of cases 36% did not have any formal education or completed less than 5^th ^grade than controls (24%). 34% cases and 26% had a family history of psychiatric illness. A similar proportion of cases and control were working. Overall household income of cases was lower than controls (Table 1).

**Table 1:** Distribution and crude association of sociodemographic factors with depression among women of reproductive age in Karachi, Pakistan

Variable	Cases n = 152	%	Controls n = 152	%	OR (95% CI)	P-Value
**Age**						0.60

15-25 years	41	27	42	27.6	1.00	

26-40 years	98	64.5	92	60.5	1.09 (0.65,1.83)	0.74

41-48 years	13	8.6	18	11.8	0.74 (0.32,1.70)	0.47

**Ethnicity**						0.40

Urdu Speaking	82	53.9	95	62.5	1.00	

Sindhi	17	11.2	14	9.2	1.40 (0.65,3.02)	0.38

Punjabi	18	11.8	19	12.5	1.09 (0.54,2.23)	0.79

Pushto	11	7.2	10	6.6	1.27 (0.51,3.15)	0.60

Balochi, Persian or Northern areas	24	15.8	14	9.8	1.98 (1.96,4.09)	0.06

**Family history of psychiatric illness**						

Yes	51	33.6	40	26.3	1.41 (0.86,2.31)	0.16

No	101	66.4	112	73.7	1.00	

**Education**						0.02

None or less than primary	54	35.5	36	23.7	1.00	

VI-XII	60	39.5	54	35.5	0.74 (0.42,1.29)	0.29

Graduation or post graduation	38	25	62	40.8	0.40 (0.22,0.73)	0.003

**Currently enrolled in school**						

Yes	6	3.9	9	5.9	0.65 (0.22,1.88)	0.43

No	146	96.1	143	94.1	1.00	

**Employed**						

Yes	18	11.8	18	11.8	1.00 (0.49,2.00)	1.00

No	134	88.2	134	88.2	1.00	

**Household**						

**No. of family members**						

≤ 5	65	42.8	90	59.2	1.00	

> 5	87	57.2	62	40.8	1.94 (1.23,3.06)	0.004

**Monthly income (Rs.)**						0.17

≤ 8000	48	31.6	32	21.1	1.00	

9000-12000	36	23.7	40	26.3	0.60(0.31,1.13)	0.11

13000-20000	43	28.3	46	30.3	0.62 (0.33,1.14)	0.12

> 20000	25	16.4	34	22.4	0.49 (0.24,0.97)	0.04

**Relation with husband**						

**Support in family conflict**	0.36					

Often	70	46.1	81	53.3	0.64 (0.33,1.24)	0.18

Sometimes	55	36.2	51	33.6	0.79 (0.40,1.59)	0.52

Never	27	17.8	20	13.2	1.00	

**Verbal, emotional or physical abuse ever**						

Yes	92	60.5	65	42.8	2.05 (1.29,3.24)	0.002

No	60	39.5	87	57.2	1.00	

**Relation with in-laws**						

**Living with in-laws**						

Yes	72	47.4	65	42.8	1.20 (0.76,1.89)	0.42

No	80	52.6	87	57.2	1.00	

**Relations**						<0.001

Good	75	49.3	122	80.3	0.07 (0.02,0.25)	<0.001

Satisfactory	52	34.2	27	17.8	0.23 (0.06,0.83)	0.02

Unsatisfactory	25	16.4	3	2	1.00	

**Verbal, emotional or physical abuse ever**						

Yes	76	50	26	17.1	4.84 (2.85,8.22)	<0.001

No	76	50	126	82.9	1.00	

61% of the cases and 43% of the controls were ever abused by spouse and 50% of the cases and 17% of the controls were ever abused by in-laws. Relationship and abuse by in-laws showed the strongest association (P- Value < 0.001).

Table 2 shows the distribution and association of various reproductive rights (right to choose partner, marital relations, right to sexuality and the right to control reproduction) among cases and controls. Cases were married at younger age (≤ 18 years of age: 39% cases vs. 22% controls) but for longer duration (≥ 5 years: 75% cases vs. 65% controls) in comparison to controls. A higher proportion of cases married on parents' decision (80% vs. 63%), were forced to married (21% vs. 6%). About 57% of cases and 45% of controls did not meet their prospective husbands before marriage. A lower proportion of cases were allowed by their husbands to initiate intimacy (59% vs. 80%).

**Table 2:** Distribution and crude association of reproductive rights with depression among women of reproductive age in Karachi, Pakistan

Variable	Cases n = 152	%	Controls n = 152	%	OR (95% CI)	*P*
**Marriage**						

**Age at marriage**						0.005

≤ 18 years	59	38.8	34	22.4	2.4 (1.40,4.10)	0.02

19-20 years	33	21.7	35	23	1.30 (0.73,2.33)	0.36

More than 20 years	60	39.5	83	54.6	1.00	

**Decision to marry**						0.001

Myself with spouse or with someone else	16	10.5	41	27	1.00	

Family member other than parents	14	9.2	15	9.9	2.39 (0.94,6.06)	0.06

Parents	122	80.3	96	63.2	3.25 (1.72,6.15)	<0.001

**Forced decision**						

Yes	33	21.7	6	3.9	6.74 (2.73,16.64)	<0.001

No	119	78.3	146	96	1.00	

**Meeting with spouse before marriage**						

Yes	66	43.4	84	55.3	0.62(0.39,0.97)	0.03

No	86	56.6	68	44.7	1.00	

**Liking for spouse before marriage**						

Yes	60	39.5	94	61.8	0.40(0.25,0.63)	<0.001

No	92	60.5	58	30.2	1.00	

**Number of years married**						

< 5	38	25	54	35.5	1.00	

≥ 5	114	75	98	64.5	1.65 (1.08,2.71)	0.04

**Satisfaction with married life**						<0.001

Very much satisfied	73	48	117	77	0.04(0.005,0.32)	0.002

Satisfied	64	42.1	34	22.4	0.12(0.016,0.99)	0.04

Not satisfied	15	9.9	1	0.1	1.00	

**Marital relations**						

**Hours/day spent with husband**						

≤ 3 hours	73	48	48	31.8	2.00 (1.25,3.19)	0.004

> 3 hours	79	52	104	68.4	1.00	

**Frequency of sexual intercourse/week**						

≤ 2	101	66.4	76	50	1.98 (1.24,3.14)	0.004

> 2	51	33.6	76	50	1.00	

**Initiation of intimacy**						

Husband	128	84.2	105	69.1	1.00	

Me or Either	24	15.8	47	30.9	0.41 (0.24,0.73)	0.002

**Husband's allowance of initiating intimacy**						

Yes	90	59.2	122	80.3	0.35 (0.21,0.59)	<0.001

No	62	40.8	30	19.7	1.00	

**Satisfaction after intimacy**						

Yes	127	83.6	147	96.7	0.17 (0.06,0.46)	0.001

No	25	16.4	5	3.3	1.00	

**Marital rape ever**						

Yes	50	32.9	19	12.5	3.43(1.90,6.17)	<0.001

No	102	67.1	133	87.5	1.00	

**Family planning decisions**						

**Family ever planned**						

Yes	78	51.3	78	51.3	1.00 (0.63,1.5)	1.00

No	74	48.7	74	48.7	1.00	

**Number of children**						

≤ 2	82	53.9	97	63.8	1.00	

> 2	70	46.1	55	36.2	1.50 (0.95,2.38)	0.08

**Decision on family planning**						0.67

Husband or family members	10	12.8	14	17.9	0.71 (0.29,1.73)	0.45

Self	9	11.5	5	6.4	1.80 (0.56,5.69)	0.31

Both	59	75.6	59	75.6	1.00	

**Decision in Disagreement**						0.35

Husband	55	70.5	45	57.7	1.39 (0.47,4.14)	0.54

Self or jointly with husband	16	20.5	25	32.5	0.73(0.22,2.41)	0.60

Physician/family members	7	9.0	8	10.3	1.00	

**Use of method of contraception**						

Yes	74	48.7	69	45.4	1.14 (0.72,1.79)	0.56

No	78	51.3	83	54.6	1.00	

**Decision on Contraception**						0.93

Husband or family members	18	21.7	15	24.3	1.00	

Self	11	14.9	11	15.9	0.83 (0.28,2.45)	0.74

Both	45	60.8	43	62.3	0.87 (0.39,1.94)	0.87

**Decision in Disagreement**						

Husband	59	79.7	47	68.1	1.84 (0.86,3.93)	0.11

Self or physician	15	20.2	22	21	1.00	

**Induced abortion**						

Yes	16	10.5	12	7.9	1.37(0.62,3.00)	0.42

No	136	89.5	140	92.1	1.00	

The final logistic regression model included, marriage decision making, age at marriage, physical, verbal or emotional abuse by in-laws, number of hours spent with husband, frequency of sexual intercourse/week and marital rape (Table 3). Factors which were significant in the univariable analysis such as education, number of family members, forced decision of marriage, meeting with spouse before marriage, liking of spouse before marriage, number of years married, satisfaction with married life, initiation or satisfaction after intimacy and husband's allowance to initiate intimacy were not significant in the multivariable analysis.

**Table 3:** Multivariable logistic regression model for association of reproductive rights and sociodemographic factors with depression among women of reproductive age in Karachi, Pakistan

Variables	Cases n (%)	Controls n (%)	Adjusted OR	95% CI
**Decision to marry**				

Myself with spouse or someone else	16 (10.5)	41 (27)	1.00	

Family member other than parents	14 (9.2)	15 (9.9)	2.45	0.79,7.5

Parents	122 (80.3)	96 (63.2)	3.51	1.67,7.37

**Age at marriage**				

≤ 18 years	59 (38.8)	34 (22.4)	2.00	1.07,3.7

19-20 years	33 (21.7)	35 (23)	1.33	0.68,2.59

> 20 years	60 (39.5)	83 (54.6)	1.00	

**Physical, verbal or emotional abuse by in-laws ever**				

Yes	76 (50)	26(17.1)	4.91	2.66,9.06

No	76 (50)	126(82.9)	1.00	

**Number of hours spent with husband**				

≤ 3 hours	73 (48)	48 (31.8)	2.33	1.34,4.08

>3 hours	79 (52)	104 (68.4)	1.00	

**Frequency of sexual intercourse/week**				

≤ 2	101 (66.4)	76 (50)	1.85	1.06,3.22

> 2	51 (33.6)	76 (50)	1.00	

**Marital rape ever**				

Yes	50 (32.9)	19 (12.5)	3.03	1.50,6.11

No	102 (67.1)	133 (87.5)	1.00	

After adjusting for the effects of other variables in the model and taking the option "myself with spouse or someone else" as the reference category, the odds of cases marrying on the decision of their parents (OR: 3.51; 95%CI: 1.67, 7.37) or on the decision of family members other than their parents were higher than controls (OR: 2.45; 95% CI: 0.79, 7.5). Odds of cases marrying at an age ≤ 18 years (OR: 2.00; 95%CI: 1.07, 3.7) or 19-20 years (OR: 1.33; 95%CI: 0.68,2.59) in comparison to > 20 years, were higher than controls. Cases were more likely to be physically, verbally or emotionally abused by in-laws than controls (OR: 4.91; 95%CI: 2.66, 9.06). Spending l ≤ 3 hours per day with spouse (OR: 2.33; 95%CI: 1.34,4.08), frequency of intercourse less than or equal to twice per week (OR: 1.85; 95%CI: 1.06,3.22) and, forced sexual relation with spouse (marital rape) were associated with depression (OR:3.03; 95%CI:1.50,6.11).

We did not find interaction between variables in the model. The Hosmer Lemshow test for goodness of fit for the final model revealed good fit (χ^2 ^= 7.72, p 0.45).

## Discussion

This is the first study to examine for the association of women's depression with violation of their reproductive rights and forms of abuse by family members in the patriarchal Pakistani society, where women's autonomy is limited. The results of this study suggest that the prevalence of domestic abuse is high and women lack various other reproductive and sexual rights, which include freedom to choose partner and marital rape, both of which are determinants of depression among Pakistani women of reproductive age. Some of the positive associations were consistent with other studies done in other parts of the world such as marital rape, [7,8] and in Pakistan such as poor relations with in-laws [13,29] and less than 15 years of age at marriage [15].

The finding that decision of marriage taken by parents (arranged marriage) is positively associated with depression is plausible because feeling of helplessness and living with an imposed partner for a lifetime could make women more vulnerable to depression, though a previous study done in Nepal had shown lower depressive scores, (though not significant) among postnatal women who had had arranged marriages [30].

Younger age at marriage predisposing to depression found in our study is consistent with a cross sectional study on postnatal Turkish women which showed a similar positive association [6]. Women who are married at a younger age tend to have spouses who are much older than them which may intensify the communication gap and power imbalance between spouses [31]. Less time spent with the husband could also be related to younger age at marriage but no previous studies were found on association of exact number of hours spent with spouse and depression, although it was found that the prevalence of domestic violence which has an association with depression [7,13], is less among couples who communicated and made joint decisions [32].

Marital rape was also found to be associated with depression, as it may lead to a feeling of degradation, negative self image and cause shame, guilt and fear which are known predisposing factors for depression. Some women with history of marital rape report flash-backs, sexual dysfunction, and emotional pain for years after the violence [9]. Marital rape may be even more depressing then rape by a stranger [33] as victims of marital rape may experience additional trauma of betrayal, but these assumptions need to be studied and explored further. There may be some under reporting of marital rape by wives in our study because it is so common, that it may be considered as a norm rather than an act of violence. In a study in Pakistan, 77% of men admitted to ever engaging in a non-consensual sex with their wives, which suggests that there is no shame or stigma attributed to the husband [34]. The fact that in our study abuse by spouse was not significant in the multivariable model could be a due to significant overlapping between emotional, verbal and physical abuse and sexual violence or marital rape which is also a form of physical violence [9,10].

Studies in the past have shown that marital rape is also associated with various gynecological diseases which may lead to lower frequency of sexual activity [8-10]. We were unable to establish a temporal relationship between low sexual activity and depression, because our study was not designed to examine this question, and depression itself leads to decreased sexual desire [24]. Other studies have shown similar associations. A study of post natal women in Taiwan and UK found an association between post natal depression and an unsatisfactory sex life [35]. Another retrospective study of low-income suburban women in Texas showed an association between depression and not having sexual intercourse in the last 3 months [36]. Further studies are required to understand this association.

Another factor found to be associated with depression was abuse by in-laws (which include mother, father and brother and sister in law), which reflects that women's mental health is affected by a family dynamic that extends beyond a marital relationship in our culture. This has already been studied in the past [13,29]. Joint family system is common in Pakistan and therefore women are more prone to physical, verbal and emotional abuse by in-laws than in other countries where nuclear families are more common and accepted culturally. Violence, abuse and relationship problems are a significant cause of depression among women and hence this finding is consistent with other studies [10,18]. Living with in laws was not associated with depression in the multivariable analysis which indicates that living in a joint family by itself is not a risk factor for depression. It may be hypothesized that married women living with supportive in laws may be resilient to depression where as an opposite effect may be the consequence of living in an abusive family.

Though some other factors such as ethnicity, being less educated, number of family members in the household, monthly income, abuse by spouse, meeting or liking spouse before marriage, forced marriage, number of years married, satisfaction with marital life, initiation of intimacy, husband allowing wife to initiate intimacy, satisfaction after intimacy and number of children were associated with depression in the univariate analysis, they did not stay so in the multivariable analysis. Some of the associations are consistent with other studies which showed a positive association of depression with being less educated, socioeconomic adversity [10,13,19,37], verbal abuse and relationship problems [19,20]. Marital conflicts, domestic violence [14,30,36], increasing duration of marriage [19] and multiparity [1] have also been shown to be associated with depression. It is a possibility that the variables like meeting spouse before marriage, forced marriage and satisfaction with marital life, initiation of intimacy and satisfaction after intimacy may have similar responses and thus mask the true associations, although the multicolinearity coefficients were found to be small.

Decision about family planning, contraception and induced abortions were not significantly associated with depression. The possible explanation can be that most of the women in this study were unaware of the meaning of family planning and the frequency of induced abortions seems to be underreported in this study, possibly due to legal [38], religious or social taboos.

Finally, 19% of the potential controls screened positive for depression, which is a high number. This reflects that women in our study population may be unaware of their depression, that they may consider their depression inappropriate to mention to a physician, or that they may experience social, cultural or economic barriers to accessing treatment for depression.

Although this study has important findings, it is also subject to certain limitations. For example, it is not possible to establish a temporal relationship in a case control study; as we cannot determine whether depression was actually preceded or followed by the associated factors. There is a possibility of selection bias for which cases and controls were selected from the same hospitals. The controls were selected from attendants accompanying the cases or any patient visiting the consulting clinics, which may have resulted in underestimation of the associations, but only 5% of the controls accompanied a case which decreases the probability of the controls having the same background as of the cases. The possibility of interviewer bias could not be completely eliminated as some subjects were not literate (6%) and hence the research officer had to fill the questionnaire.

The tools used to measure verbal, emotional and physical abuse were not validated but the questions asked for measuring verbal, emotional and physical abuse have been used in WHO multicountry study [10]. The reason for not using validated tools was that no tools for measuring abuse have been validated in Pakistan and validating tools was not in the scope of this study. This could have lead to imprecise measure of some exposure variables and hence to the finding, that abuse by spouse was not found to be associated with depression in the multivariable analysis.

100% of the participants answered all the questions with a 96% participation rate, still there are chances that subjects may not have revealed the true response to profoundly personal questions. The generalizability of results of a hospital based study to the general population is also limited. Larger community based studies that use probability sampling of subjects are required to substantiate our findings.

## Conclusion

Our study found significant associations in the Pakistani women between depression and lack of some reproductive rights as manifested by: being under 18 years of age at marriage; decisions of marriage being determined by parents; and marital rape. Also significantly associated with depression were abuse by in-laws, less time spent daily with husband and lower frequency of intercourse.

Our study indicates that there is a need to focus on the protection of reproductive rights of women in our society. These findings have important policy implications for reducing morbidity level from highly prevalent depression among women. Knowledge and appreciation of lack of autonomy in reproductive matters and its association with depression could possibly make a difference in reducing the incidence of depression among women, which is high in Pakistan. Families and communities should be educated regarding the importance of women's autonomy in her marriage decision. Women should be made aware of their reproductive and sexual rights, and married women should be asked screening questions regarding domestic abuse and marital rape. Clinicians can support positive mental health outcomes through early identification of women who may be at risk for psychological distress as a result of domestic violence and denial of other reproductive rights and could refer them for individual or marital counseling. Especially in couples where there is communication problem, physicians can help to provide counseling to bridge this gap. Primary care physicians should be trained to identify depression and provide appropriate guidance and counseling not only to the women at risk but also to their families about the predisposing factors for depression, particularly the reproductive rights, which is in fact a human right.

## Competing interests

The authors declare that they have no competing interests.

## Authors' contributions

All the authors have read and approved the manuscript. FAA is the principal investigator and has contributed in development of protocol, ERC approval, data collection, data analysis, manuscript writing and submission. SMI has supervised the whole project and was involved in development of protocol and thesis write up. BSA was the clinical supervisor of the project and has contributed in conceiving the idea of this project, protocol development and thesis write-up. NZJ was the committee member and has made major contribution in data analysis, thesis write up and preparation of manuscript.

## Pre-publication history

The pre-publication history for this paper can be accessed here:

http://www.biomedcentral.com/1471-244X/9/77/prepub

## References

[B1] UNDP Summary of the programme of action International Conference on Population and Development. Cairo 1994

[B2] World Association for Sexology Declaration of Sexual Rights14th world congress of sexology, Hong Kong 1999

[B3] Manzoor K An attempt to measure female status in Pakistan and its impact on reproductive behaviour Pak Dev Rev 1993 32 917 27 12346816

[B4] Adolescent Reproductive and Sexual Health An Explorationof Trends in Pakistan 2000 Pakistan Voluntary Health and Nutrition Association [PAVH NA]

[B5] O'Campo E Muntaner C Labor market experience, work organization, gender inequalities and health status: results from a prospective analysis of US employed women Soc Sci Med 2004 58 3 585 94 10.1016/S0277-9536(03)00230-2 14652054

[B6] Inandi T Elci OC Ozturk A Egri M Polat A Sahin TK Risk factors for depression in postnatal first year, in Eastern Turkey International Journal of Epidemiology 2002 31 1201 07 10.1093/ije/31.6.1201 12540723

[B7] Dienemann J Boyle E Baker D Resnick W Wiederhoen N Campbell JC Intimate partner abuse among women diagnosed with depression Issues in Mental Health Nursing 2000 21 5 499 513 10.1080/01612840050044258 11261074

[B8] Campbell J Health consequences of intimate partner violence The Lancet 2002 359 1331 6 10.1016/S0140-6736(02)08336-8 11965295

[B9] Bergen RK New Research and Directions 2006 The National Online Resource Center on Violence Against Women

[B10] García-Moreno C Jansen HAFM Ellsberg M Heise L Watts C WHO Multi-country Study on Women's Health and Domestic Violence against Women Initial results on prevalence, health outcomes and women's responses 2005 Geneva: World Health Organization

[B11] Malik N Women's protection bill is not enough Chowk 2007

[B12] WHO Mental health aspects of women's reproductive health. A global review of the literature 2009 Geneva: World Health Organization

[B13] Ali BS Rahbar MH Naeem S Tareen AL Gul A Samad L Prevalence of and factors associated with anxiety and depression among women in a lower middle class semi-urban community of Karachi, Pakistan J Pak Med Assoc 2002 52 11 513 7 12585371

[B14] Tareen E The perception of social support and the experience of depression in Pakistani women (Phd thesis) 2000 Colchester: University of Essex

[B15] Fikree FF Bhatti LI Domestic violence and health of Pakistani women Int J Gynaecol Obstet 1999 65 2 195 201 10.1016/S0020-7292(99)00035-1 10405066

[B16] Fikree FF Razzak JA Durocher J Attitudes of Pakistani men to domestic violence: a study from Karachi, Pakistan JMHG 2005 2 1 49 58

[B17] El-Zanaty FH Hussein EM Shawky GA Way AA Kishor S Egypt Demographic and Health Survey 1995

[B18] UNFPA State of world population 2000 2000

[B19] Ilyas M Rachel J Risk factors, prevalence, and treatment of anxiety and depressive disorders in Pakistan: Systematic review BMJ 2004 328 794 8 10.1136/bmj.328.7443.794 15070634 PMC383372

[B20] Amin A Gadit M Mugford G Prevalence of Depression among Households in Three Capital Cities of Pakistan: Need to Revise the Mental Health Policy Plos one 2007 2 2 10.1371/journal.pone.0001274 PMC1790700 17299589

[B21] Dodani S Zuberi RW Psychiatric disorders in the northern areas of Pakistan J Pak Med Assoc 2000 50 137 11242709

[B22] Husain N Gater R Tomenson B Creed F Social factors associated with chronic depression among a population-based sample of women in rural Pakistan Social Psychiatry and Psychiatric Epidemiology 2004 39 8 618 624 10.1007/s00127-004-0781-1 15300372

[B23] Husain N Creed F Tomenson B Depression and social stress in Pakistan Psychol Med 2000 30 2 395 402 10.1017/S0033291700001707 10824659

[B24] Subhash CB Shashi KB Depression in women: diagnostic and treatment considerations AAFP 1999 60 225 40 10414640

[B25] A user's guide to self-reporting questionnaire (SRQ) 1994 Geneva: World Health Organization

[B26] Husain N Gater R Tomenson B Creed F Comparison of the Personal Health Questionnaire and the Self Reporting Questionnaire in rural Pakistan J Pak Med Assoc 2006 56 8 366 370 16967789

[B27] Tinker GA Improving women's health in Pakistan. Karachi 1999 World Bank

[B28] Schlesselman JJ Case-control studies. Design, conduct, analysis 1982 New York, Oxford University Press

[B29] Green K Broome H Mirabella J Postnatal depression among mothers in the United Arab Emirates: socio-cultural and physical factors Psychol Health Med 2006 11 4 425 31 10.1080/13548500600678164 17129919

[B30] Ho-Yen SD Bondevik GT Eberhard-Gran M Bjorvatn B Factors associated with depressive symptoms among postnatal women in Nepal Acta Obstet Gynecol Scand 2007 86 3 291 7 10.1080/00016340601110812 17364302

[B31] Complex Role for Marriage in HIV Risk 2007 Population Briefs

[B32] Hindin Michelle J Linda SA Women's Autonomy, Men's Autonomy and Gender Violence in the Philippines: The Case for Promoting Couple Communication 2000 Annual Meeting of the Population Association of America, Los Angeles, California

[B33] Plichta SB Falik M Prevalence of violence and its implications for women's health Women's Health Issues 2001 11 244 258 10.1016/S1049-3867(01)00085-8 11336864

[B34] Huang YC Mathers NJ A comparison of sexual satisfaction and post-natal depression in the UK and Taiwan Int Nurs Rev 2006 53 3 197 204 10.1111/j.1466-7657.2006.00459.x 16879182

[B35] Berenson AB Brietkopf CR Wu ZH Reproductive correlates of depressive symptoms among low- income minority women Obstet Gynecol 2003 102 6 1310 17 10.1016/j.obstetgynecol.2003.08.012 14662220

[B36] Husain N Chaudhry I Afridi MA Tomenson B Creed F Life stress and depression in a tribal area of Pakistan Br J Psychiatry 2007 190 36 41 10.1192/bjp.bp.106.022913 17197654

[B37] Abortion Policies A Global Review 2001 The United Nations Department of Economic and Social Affairs, Population Division

[B38] Shaikh MA Domestic violence against women-perspective from Pakistan J Pak Med Assoc 2000 50 9 312 4 11043022

